# Tumourigenesis Driven by the Human Papillomavirus Type 16 Asian-American E6 Variant in a Three-Dimensional Keratinocyte Model

**DOI:** 10.1371/journal.pone.0101540

**Published:** 2014-07-01

**Authors:** Robert Jackson, Melissa Togtema, Paul F. Lambert, Ingeborg Zehbe

**Affiliations:** 1 Probe Development & Biomarker Exploration, Thunder Bay Regional Research Institute, Thunder Bay, Ontario, Canada; 2 Department of Biology, Lakehead University, Thunder Bay, Ontario, Canada; 3 McArdle Laboratory for Cancer Research, University of Wisconsin School of Medicine and Public Health, Madison, Wisconsin, United States of America; Albert Einstein College of Medicine, United States of America

## Abstract

Infection with a transforming human papillomavirus (HPV) such as type 16 (of species *Alphapapillomavirus* 9) causes ano-genital and oral tumours via viral persistence in human squamous cell epithelia. Epidemiological studies showed that the naturally occurring HPV16 Asian-American (AA) variant (sublineage D2/D3) is found more often than the European Prototype (EP) (sublineage A1) in high-grade cervical neoplasia and tumours compared to non-cancer controls. Just three amino acid changes within the early gene, E6, of HPV16 AA have been linked to this augmented tumourigenicity. The AAE6 variant's greater immortalizing and transforming potential over EPE6 has recently been confirmed in retrovirally-transduced keratinocytes expressing the E6 gene only. However, the tumourigenic role of the full-length viral genome of HPV16 has not yet been addressed with regard to these E6 variants. To investigate this process in the context of these two HPV16 E6 genotypes, an organotypic tissue culture model was used to simulate the HPV infectious life cycle. The AAE6 variant demonstrated an enhanced ability over EPE6 to drive the viral life cycle toward tumourigenesis, as evidenced phenotypically—by a more severe grade of epithelial dysplasia with higher proliferation and deregulated differentiation, and molecularly—by high viral oncogene E6 and E7 expression, but lack of productive viral life cycle markers. In contrast, EPE6 had low E6 and E7 but high E1∧E4 expression, indicative of a productive life cycle. We suggest increased viral integration into the host genome for AAE6 as one possible mechanism for these observed differences from EPE6. Additionally, we found downstream effects on immortalization and host innate immune evasion. This study highlights how minor genomic variations in transforming viruses can have a significant affect on their tumourigenic ability.

## Introduction

Human papillomaviruses (HPVs) are double-stranded DNA viruses that infect skin basal keratinocytes, as well as genital and upper digestive tract mucosa [1]. HPVs are classified as low-risk and high-risk, based on tumour-forming potential [2]. HPV type 16, of species *Alphapapillomavirus* 9, is the most prevalent of the high-risk types and is causally linked to ano-genital (i.e., cervical, vulvar, and anal) as well as tumours of the head and neck [3], making it an important subject for study [4]. Like other transforming DNA viruses, HPV16 encodes viral oncoproteins, which act synergistically in infected keratinocytes [5]. Two intracellular oncoproteins, E6 and E7, play an important role in the immortalization and malignant transformation of HPV-infected cells [6]. E7 induces increased cellular proliferation by binding to and inactivating the tumour suppressor retinoblastoma protein. This releases a transcription factor (E2F) and allows the HPV-infected cell to replicate, even in the absence of growth factors [7]. For a recent review of the E7 protein, see Roman and Münger [8]. E6 maintains cellular immortalization and transformation, and upholds tumour growth [9]. These activities are mediated by E6's ability to promote telomerase activity and degrade cellular proteins, such as the tumour suppressor p53, via protein-protein interactions [10]. E6 has many additional cellular binding partners due to its two zinc-binding domains as well as its PDZ-binding domain. For a recent review of the E6 oncoprotein, see Vande Pol and Klingelhutz [11].

Not all high-risk HPV infections lead to tumours and manifestations of malignant transformations may be attributed to specific HPV genome variants [12]. Variants of distinct phylogenetic branches have been described, based on their geographic distribution and origin of discovery [13], [14]. The Asian-American (AA) variant (of sublineage D2 and D3, GenBank accession numbers AY686579 and AF402678, respectively) is one such example, containing six single nucleotide polymorphisms in the E6 gene when compared to the European Prototype (EP) reference sequence (of sublineage A1, GenBank accession number K02718) [15]–[17]. Three of these polymorphisms are non-synonymous, resulting in the following amino acid changes in the 151-residue AAE6 protein translated from the second start codon: Q14H/H78Y/L83V. Variant-specific tumourigenic risk has been reported for high-grade cervical intraepithelial neoplasias (CIN 2/3) and tumours in American populations [18]–[21]. Women with non-E (including AA) HPV16 variants had a 4.5 times higher risk of developing CIN 2/3 than women with E variants [18]. As well, in women initially without CIN 2/3, the risk of developing CIN 3 was three times higher for women with AA compared to E variants [21]. The HPV16 AA variant has also been associated with a higher risk of cervical cancer than E variants; the risk of cervical cancer was eight times higher for patients with AA compared to E variants, when compared to a non-cancer control group [20]. From the same study, there is also evidence that AA variants are associated with significantly earlier cancer occurrence (case patients with AA were 7.7 years younger than those with E variants) [20]. Additionally, epidemiological studies of European populations by our group have shown that AA and other non-EP HPV16 E6 variants are over-represented in cervical tumours [22]–[24].

To dissect previous epidemiology findings, our group has been investigating the role of E6 variants on oncogenic potential. Previously, we used an *in vitro* model that mimics a persistently-infected and post-integrated keratinocyte system [25]–[27], based on primary human foreskin keratinocytes (PHFKs) and normal (or near-diploid) immortalized keratinocytes (NIKS) [28]. Using retrovirally-transduced PHFKs, we have recently demonstrated the enhanced *in vitro* oncogenic abilities of AAE6 alone over EPE6, specifically at promoting cellular immortalization, undergoing transformation to resilient phenotypes, and promoting migration and invasiveness (although both variants were able to degrade p53 and enhance hTERT expression similarly) [26], [27]. However, there still exists a significant knowledge gap: the full-length HPV16 genomes, containing either the EPE6 or AAE6 gene, have not been studied in the context of early tumourigenesis for initial viral life cycle effects in an early infection scenario. Given that the majority of HPV infections are normally cleared [2], there is justification to consider that increased risk of the AAE6 variant for high-grade CIN and cervical tumours may be due to its ability to persist through unique or augmented viral life cycle effects occurring early within host cells, in the context of the full-length HPV16 genome.

In the current study, we determined the abilities of two HPV16 E6 genotypes, EPE6 and AAE6, to contribute to the tumourigenic process in host epithelium. We achieved this by using a full-length HPV16 genome containing either EPE6 or AAE6 in the context of an organotypic, three-dimensional model of keratinocyte epithelium (the required host environment for an HPV infection). We show that AAE6 has enhanced abilities over EPE6 in driving the infected epithelium toward tumourigenesis, as phenotypically manifested by advanced dysplastic growth and abrogated differentiation, and molecularly by increased viral oncogene expression and host changes potentially conferring viral persistence.

## Results and Discussion

### HPV16-containing rafts resembled squamous cell dysplasia of different grades between EPE6 and AAE6

Our previous investigations explored the tumourigenic role of HPV16 E6 variants in the context of the E6 gene alone (and not the whole viral genome) in raft [25], [26] and monolayer cultures [27]. We reported that AAE6 exceeded EPE6 in proliferation efficiency with an underlying mechanism attributable to the Warburg effect, the metabolic phenomenon observed in transformed cells [26], [27]. This offered one possible molecular explanation of the epidemiological findings of AA being a higher risk factor for high-grade CIN and cervical tumours than EP.

In the current study, we analyzed EPE6 and AAE6 in the context of their full-length HPV16 genomes, within a model of their natural host tissue: stratified squamous epithelium. We accomplished this using an established three dimensional, organotypic tissue culture model [25], [29] that can reproducibly form *in vivo*-like dermal and epidermal layers and be used to study the productive viral life cycle and HPV-induced tumourigenesis.

A key feature of our squamous epithelium model is the use of NIKS, an immortalized human foreskin keratinocyte cell line that undergoes terminal differentiation when grown as rafts [28]. We therefore used raft cultures of HPV-transfected NIKS for our studies. We reasoned that this system should mimic the neoplastic progression of HPV-infected skin cells. Epithelial differentiation is essential for the productive HPV viral life cycle to take place [29]. During an *in vivo* infection in a human host, HPV genomes are not found in every keratinocyte of the stratified squamous epithelium. Instead, HPV infection can only be evidenced focally [30], [31]. In creating our keratinocyte model, we utilized a transfection method we had developed earlier [29] (described in detail in Materials and Methods), which met these requirements. This method generates a model that is comparable to *in vivo* and useful for studying the differences between variant genomes. For raft experiments, we conducted three independent experiments with identically paired-passage NIKS (passages 4–6 after transfection) of each E6 genotype to ensure the highest possible experimental reproducibility.

**Figure 1:**
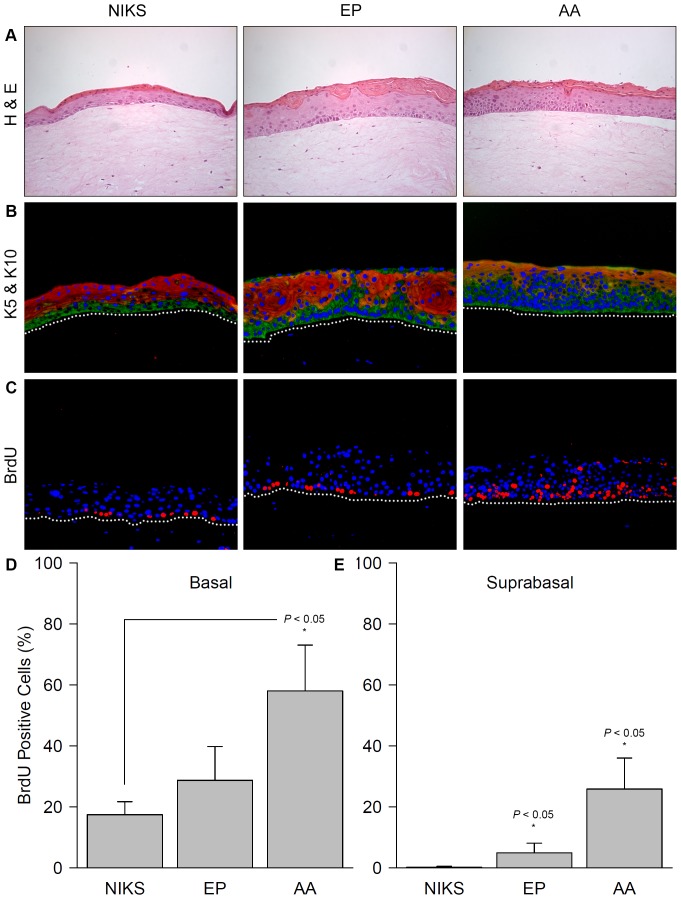
Phenotypic characterization of the three-dimensional epithelial model: morphology, differentiation pattern, and proliferation status. (A) Haematoxylin and eosin staining of sectioned formalin-fixed paraffin-embedded European Prototype (EP) HPV16 E6 variant (EPE6) and Asian-American (AA) HPV16 E6 variant (AAE6) raft cultures (200× magnification). (B) Immunofluorescent overlay micrograph of sectioned formalin-fixed paraffin-embedded raft cultures (200× magnification). Green fluorescence represents basal keratinocyte marker keratin 5 (K5), red represents suprabasal cell marker keratin 10 (K10), and blue DAPI staining indicates nuclei; the white-dotted line represents the basal membrane. (C) Immunofluorescent overlay micrograph of sectioned formalin-fixed paraffin-embedded raft cultures treated with bromo-2-deoxyuridine (BrdU) (200× magnification). Red fluorescence represents proliferation marker BrdU, incorporated into proliferating cells' nuclei; the white-dotted line represents the basal membrane. (D, E) Quantification of basal and suprabasal BrdU positive cells as a percentage of total number of DAPI-stained nuclei in each compartment. Data are presented as means, while error bars represent SD. Statistical analyses performed were: a Kruskal-Wallis test followed by pair-wise Wilcoxon rank-sum post-hoc with Bonferroni correction (*n* = 5).

Squamous cell neoplasias of the skin, the ano-genital and upper digestive tract arise from precursor lesions displaying mild to severe epithelial changes with an increase in epithelial layers (hyperplasia) and nuclear atypia. As the lesion evolves, there is progressive involvement of epithelial layers until they are fully replaced by atypical cells no longer exhibiting differentiated properties. After 14 days (an established time span for HPV life cycle studies [32]), our rafts had fully differentiated epithelia on the distal perimeter of each circular culture (Figure 1A). The untransfected NIKS epithelium appeared to be the thinnest, resembling normal skin with a clearly discernable undifferentiated basal layer and three differentiated suprabasal layers. The HPV16-positive cultures had thicker epithelia, each demonstrating hyperplasia (increased number of layers compared to normal skin) with atypical cells. The EPE6 epithelium had, on average, six layers—with one to two layers of basal-like, undifferentiated cells followed by four to five layers of larger (differentiated) suprabasal-like cells. Hyperplasia in AAE6 was seen to a greater degree than with EPE6, with up to eight layers of basal-like, undifferentiated cells that were partially interspersed with suprabasal-like cells. Squamous keratinized cells (corresponding to *stratum granulosum* and *lucidum*) with pyknotic nuclei in the uppermost layers were seen in both epithelia. Thus, the EPE6 epithelium resembled a very mild dysplasia, while the AAE6 epithelium demonstrated morphological changes reminiscent of moderately dysplastic epithelium, as assessed by a histopathologist (Dr. Nicholas Escott, personal communication).

### Markers of early squamous cell tumourigenesis were elevated in AAE6 *versus* EPE6 rafts

A premature migration of proliferating keratinocytes from the basal into the suprabasal epithelial compartment is a hallmark of early squamous cell tumourigenesis [33]–[35]. Such dysregulated epithelial differentiation is mediated by the HPV oncogenes E6 and E7 during HPV-related tumourigenesis [36]. To characterize the E6 variant-specific effects in our raft model and to strengthen our histological findings, immunofluorescence to stain keratin 5 (K5) was used as a basal and keratin 10 (K10) as a suprabasal keratinocyte differentiation marker [25]. Based on the histological results above, we expected both HPV16-containing rafts to have differential staining patterns with a disrupted K5 and K10 phenotype compared to HPV-negative tissue and to each other. Control rafts demonstrated a phenotype reminiscent of normal skin, with K5 present in the basal layer of the raft epithelium (proliferating compartment characterized by cells that still have the ability to enter the cell cycle) and K10 in all subsequent suprabasal layers (differentiating compartment characterized by terminally differentiated keratinocytes that have lost the ability to enter the cell cycle) (Figure 1B). In contrast, within the HPV16-positive cultures, K5 and K10 were aberrantly expressed, as expected in early squamous cell tumourigenesis [33]–[35]. The suprabasal zone was almost entirely invaded by K5-positive keratinocytes within AAE6 in more than half of the epithelial thickness, but only focally in EPE6 cultures; in control rafts, 3/4 of the epithelium stained with K10 (Figure 1B). Consequently, the suprabasal compartment showed disrupted and diminished K10 staining, but enhanced K5 staining in both E6 rafts and this was more pronounced in the AAE6 rafts (Figure 1B). The K5/K10 ratio was calculated for all rafts to quantitatively assess a shift from a normal (K5/K10 of NIKS) to an increasingly basal phenotype (K5/K10 > NIKS): NIKS  = 0.61 +/− 0.06, EPE6  = 0.86 +/− 0.31, and AAE6  = 1.25 +/− 0.33 (Figure S1) with an overall significant difference among the three means (*P*<0.05) and an increasing basal phenotype in EPE6 notable even more so in AAE6.

Elevated proliferation driven by oncogenes is an early step in tumour formation and eventually leads to chromosomal instability [37]. HPV16 is known to promote suprabasal proliferation of the squamous epithelium that it typically infects [38] thereby causing epithelial hyperplasia: we showed this for both EPE6 and AAE6 rafts (Figure 1). In addition, our previous work in monolayer primary keratinocytes demonstrated that AAE6-transduced cultures undergo population doublings significantly faster than do EPE6-transduced cultures, demonstrating the higher proliferation efficiency of the AAE6 variant [26], [27]. To quantitatively determine the extent of cellular proliferation in the basal and suprabasal compartments of the raft cultures, the thymidine analog, BrdU, was added to culture media 24 hours before harvesting, allowing proliferating cells to incorporate it into their DNA (Figure 1C). Although the proportion of proliferating basal cells in AAE6-infected NIKS (58.04 +/− 15.00%) was not significantly higher than in EPE6 (28.70 +/− 11.09%, *P*>0.05), AAE6 basal proliferation was significantly higher than HPV-negative NIKS (17.43 +/− 4.28%, *P*<0.05) (Figure 1D). The percentage of proliferating basal cells in EPE6-infected NIKS was not significantly higher than in uninfected NIKS (*P*>0.05). Suprabasal proliferation, on the other hand, was significantly higher than HPV-negative NIKS (0.16 +/− 0.36%), in both EPE6 (4.94 +/− 3.20%, *P*<0.05) and in AAE6 (25.85 +/− 10.17%, *P*<0.05) (Figure 1E). Furthermore, suprabasal proliferation in AAE6 was significantly higher than in EPE6 (*P*<0.05). While both E6 cultures demonstrated increased suprabasal BrdU incorporation over uninfected NIKS, AAE6 showed the greatest overall proliferation compared to NIKS and to EPE6, suggesting that the AAE6 life cycle uniquely enhances host DNA synthesis.

**Figure 2:**
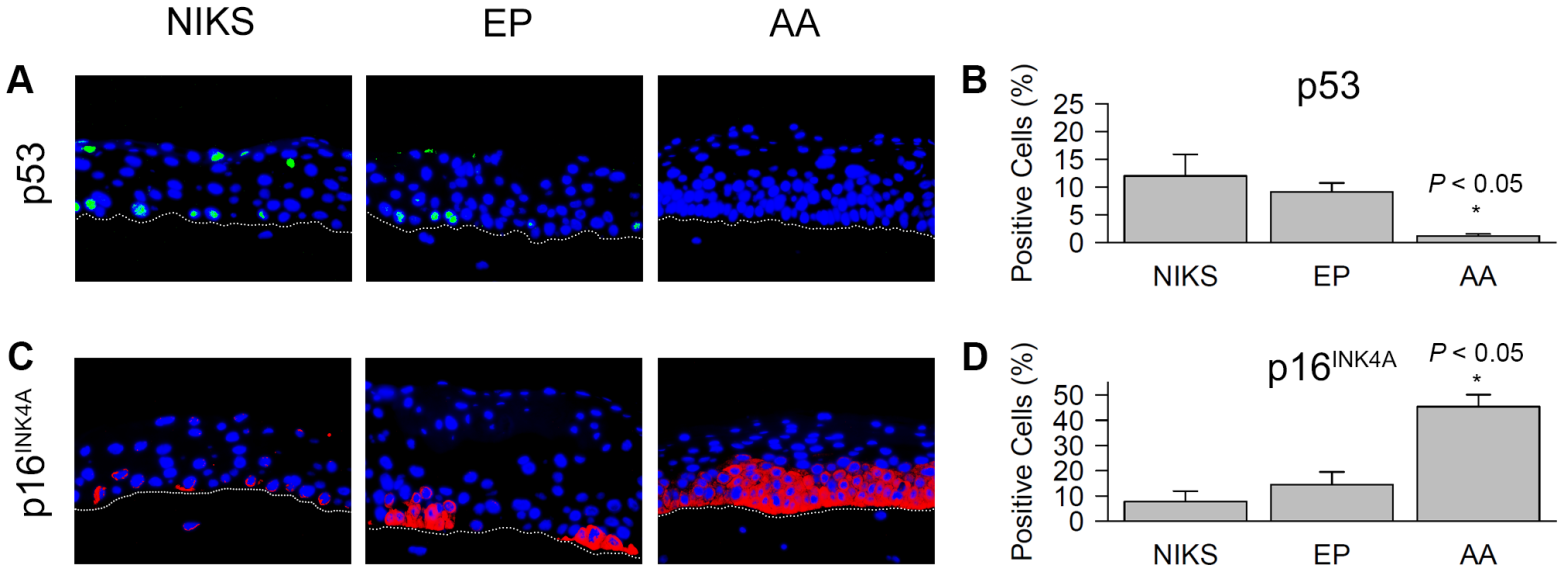
Apoptosis and cell cycle markers in HPV16-infected three-dimensional epithelia. Immunofluorescent overlay micrographs of sectioned formalin-fixed paraffin-embedded European Prototype (EP) HPV16 E6 variant (EPE6) and Asian-American (AA) HPV16 E6 variant (AAE6) raft cultures (400× magnification). Nuclei are indicated by blue DAPI staining; the white-dotted line represents the basal membrane. Total positive cells were quantified as a percentage of total number of DAPI-stained nuclei. (**A**) Green nuclear fluorescence represents p53. (**B**) Quantification of p53-positive cells. (**C**) Red diffuse cytoplasmic and nuclear fluorescence represents p16^INK4A^. (**D**) Quantification of p16^INK4A^-positive cells. Data are presented as means, while error bars represent SD. Statistical analyses performed were: a Kruskal-Wallis test followed by pair-wise Wilcoxon rank-sum post-hoc with Bonferroni correction (*n* = 5).

Intrinsic apoptotic mechanisms, such as those supported by tumour suppressor p53, exist within cells to prevent aberrant growth. Given the anti-apoptotic ability of E6 and its contribution to tumourigenesis, we assessed p53 protein levels in raft epithelia by immunofluorescence (Figure 2A). In AAE6 rafts (1.21 +/− 0.35%), the percentage of p53-positive cells was significantly lower than in NIKS (12.01 +/− 3.89%, *P*<0.05) and EPE6 (9.10 +/− 1.65%, *P*<0.05) (Figure 2B). Notably, EPE6 was not significantly different from NIKS (*P*>0.05). Although the percentage of p53-positive cells is not significantly lower in EPE6 compared to NIKS control cultures, we still expect that EPE6 protein was degrading p53. Heterogeneous viral presence, and therefore E6 expression, among host cells is likely responsible for a lack of discernable p53 degradation at the two week time point when analyzing a raft section. Given these limitations, our observation that p53-positivity is lowest in AAE6 is noteworthy. Hence we see a trend showing a decreasing amount of p53 with increased severity of the HPV+ lesion as seen in previous reports [39], [40].

While E6 disrupts p53 and has a cumulative anti-apoptotic effect on host cells, E7 is known to disrupt pRb and other cell cycle related markers. One such marker, p16^INK4A^, is over-expressed as a result of increased E7 expression in epithelium [41]. Strong diffuse staining of cytoplasm and nuclei was observed primarily in AAE6 rafts (Figure 2C). The percentage of p16^INK4A^-positive cells was significantly higher in AAE6 (45.37 +/− 4.78%) compared to HPV-negative NIKS (7.80 +/− 4.08%, *P*<0.05) and EPE6 (14.51 +/− 4.99%, *P*<0.05) (Figure 2D). Expression of p16^INK4A^ by EPE6 was not significantly different from NIKS (*P*>0.05). The lower p16^INK4A^ levels in EPE6 are to be expected since the severity of the lesion is considerably less than that of AAE6. There is literature evidence to support an increase in p16^INK4A^ as lesion severity increases [41]–[45], and we expected this was due to increased E7 expression in AAE6 rafts.

We have shown that AAE6 (as compared to EPE6) manifests a moderately dysplastic epithelium due to elevated suprabasal proliferation (increased BrdU incorporation), exhibits dysplastic markers (increased and diffuse p16^INK4A^ with decreased p53 staining) and experiences aberrant migration of cycling cells from the proliferating to the differentiating compartment (more layers of undifferentiated suprabasal cells and more extensive K5 expression). Together, these results strongly suggest that AAE6 has increased tumourigenic ability compared to EPE6.

### AAE6 demonstrated elevated expression of E6/E7 oncogenes, but reduced expression of viral life cycle markers compared to EPE6

An active viral life cycle is a major factor contributing to viral persistence in the HPV-infected host. Typical markers of the HPV life cycle include the E1∧E4 spliced transcript [46] and L2, along with L1, which are late-stage encapsidation proteins [47]. While E6 and E7 are oncogenes that are perpetually expressed during a typical viral life cycle, their expression levels are kept low by their modulator, E2 [48]–[58].

**Figure 3:**
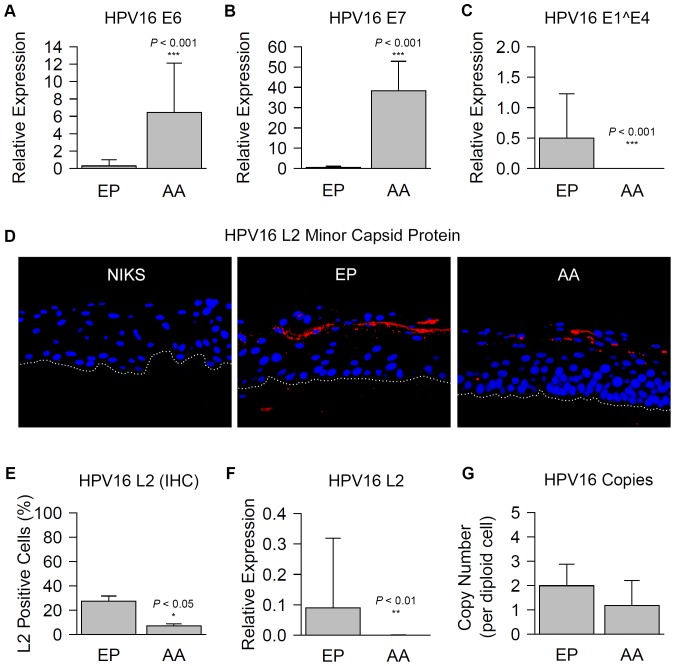
Characterization of HPV16 viral expression and production in three-dimensional epithelia. Viral expression and production in European Prototype (EP) HPV16 E6 variant (EPE6) and Asian-American (AA) HPV16 E6 variant (AAE6) raft cultures. (**A, B, C**) Relative expression of E6, E7, and E1∧E4 transcripts by qRT-PCR was calculated by the modified Livak method (2-^ΔCt^), since NIKS calibrator sample had zero viral gene expression. HPRT1 was used a reference gene. (**D**) Immunofluorescent overlay micrograph of sectioned formalin-fixed paraffin-embedded raft cultures (400× magnification). Red fluorescence represents HPV16 L2 in the superficial zone of epithelium. Blue fluorescence represents nuclear-staining by DAPI. White-dotted line represents the basal membrane. (**E**) Quantification of L2 positive cells as a percentage of total number of DAPI-stained nuclei. (**F**) Relative expression of L2 transcript, calculated as described above. (**G**) HPV16 copies per diploid cell as determined by copy number assay. Data are presented as means, while error bars represent SD. Statistical analyses of differences between EPE6 and AAE6 were performed by Wilcoxon rank-sum tests in all cases (*n* = 9 for transcription data, *n* = 4 for IHC quantification, and *n* = 5 for genome copy numbers).

Relative levels of E6 and E7 expression, quantified through qRT-PCR, followed a variant-specific pattern indicative of proliferation and protein expression of dysplastic markers p53 and p16^INK4A^. E6 and E7 expression by AAE6 (6.44 +/− 5.68 and 38.31 +/− 14.55, respectively) was significantly higher than EPE6 (0.29 +/− 0.71 and 0.43 +/− 0.67, respectively) after 14 days of raft culturing (*P*<0.001) (Figure 3A, B). In contrast, the E1∧E4 spliced transcript expression by EPE6 (0.50 +/− 0.73) was significantly higher than AAE6 (7.67×10^−5^ +/− 6.35×10^−5^, *P*<0.001) (Figure 3C). In line with this observation, there appeared to be less capsid protein in rafts positive for AAE6 compared to EPE6, as evidenced by L2 immunofluorescence (Figure 3D). Indeed, there were significantly fewer L2-positive cells in AAE6 (7.09 +/− 1.76%) than EPE6 (27.43 +/− 4.26%, *P*<0.05) (Figure 3E), which paralleled significantly lower L2 transcription in AAE6 (3.85×10^−4^ +/− 4.01×10^−4^) compared to EPE6 (0.09 +/− 0.23, *P*<0.01) (Figure 3F). Notably, these differences in viral gene expression were not caused by a difference in HPV16 copy number per diploid cell in the raft cultures (Figure 3G), which was not significantly different between EPE6 (1.99 +/− 0.89 copies) and AAE6 (1.18 +/− 1.03 copies, *P*>0.05) cultures.

The surprising difference between viral expression patterns in our EPE6 and AAE6 rafts, supported by previous studies demonstrating HPV16 integration [54], [59], may suggest that AAE6 tends to integrate into host cell genomes, while EPE6 may maintain an episomal status in our raft culture model. This is further emphasized by the fact that after 21 (rather than 14) days in culture, this pattern was unchanged for AAE6 but even more pronounced in EPE6, when its E1∧E4 levels peaked [unpublished data]. An integration scenario is associated with but not necessarily required for HPV-driven tumourigenesis—HPV DNA integration into host genomic DNA is a recognized phenomenon [60]. Indeed, viral DNA integration has been reported as a way of viral persistence for the adeno-associated parvovirus [61], sequences of which have been found endogenized into host genomes, including humans [62].

### Loss of E2 with increased E6/E7 oncogene expression and host immune evasion by AAE6

**Figure 4:**
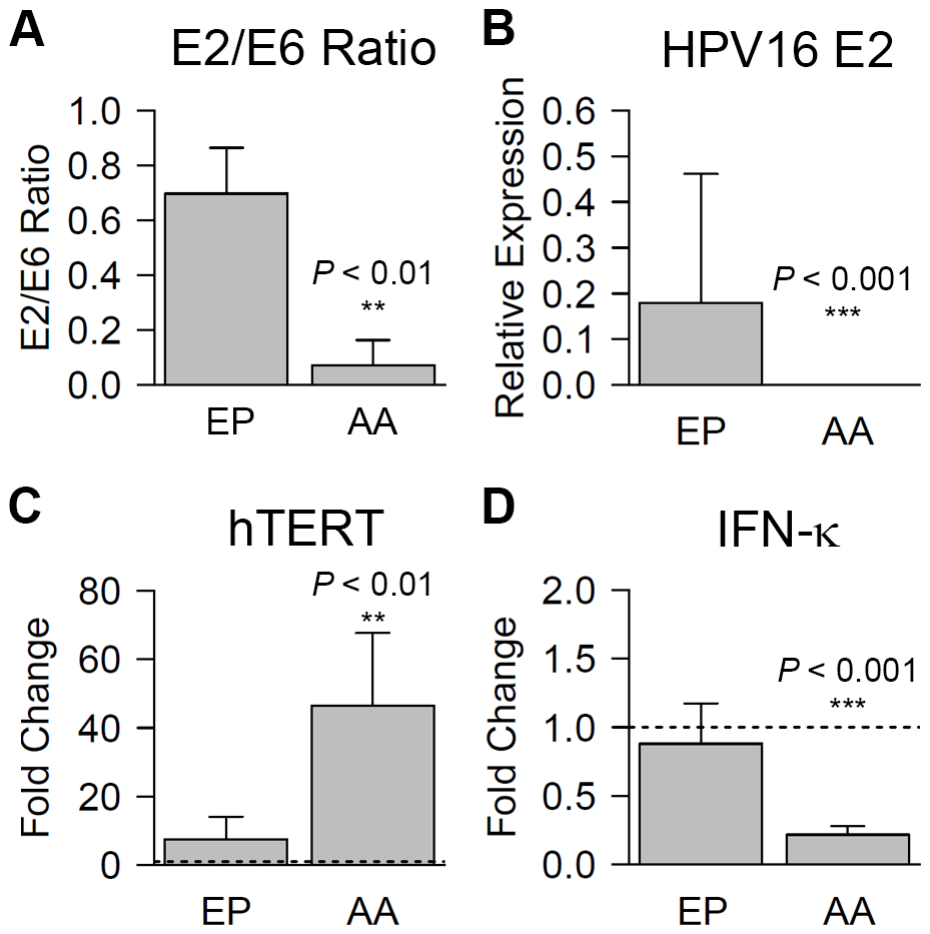
AAE6 has lower E2/E6 ratio and E2 expression, with enhanced oncogene-dysregulation and downstream effects on hTERT and IFN-κ. (A) E2/E6 ratio in European Prototype (EP) HPV16 E6 variant (EPE6) and Asian-American (AA) HPV16 E6 variant (AAE6) raft cultures. (B) Relative expression of E2 transcript was calculated by the modified Livak method (2-^ΔCt^) since NIKS calibrator sample had zero viral gene expression. HPRT1 was used a reference gene. (C) hTERT fold change was calculated by the Livak method (2^-ΔΔCt^, HPRT1 as reference gene, and NIKS as calibrator represented by the dotted line at fold change  = 1). (D) IFN-κ fold change was calculated by the Livak method (2^−ΔΔCt^, HPRT1 as reference gene, and NIKS as calibrator represented by the dotted line at fold change  = 1). Data are presented as means, while error bars represent SD. Statistical analyses of differences between EP and AA were performed by Wilcoxon rank-sum tests in all cases (*n* = 5 for E2/E6 ratio and *n* = 9 for transcription data).

The distinct increase of E6/E7 and decrease of E1∧E4 in AAE6 raft cultures prompted us to investigate the physical status of the HPV16 genome within our host keratinocyte rafts. Additionally, AAE6 rafts demonstrated histological differences that were reminiscent of higher grade cervical lesions phenotypically similar to results reported by others [63], [64]. Given that E2 interruption may be an indication of integration and loss of viral episomes [65]–[67], resulting in dysregulation of E6 and E7 [48]–[58], we employed a rapid and sensitive qPCR-based method to measure E2-disruption. This quantitative technique has been compared alongside Southern blot [68], [69], and although specific for only detecting hinge-region E2-disruptions, was a useful tentative method for us due to its low DNA input requirements and application to both variants yielding reproducible differences. The AAE6 rafts were found to have a significantly lower E2/E6 ratio (0.07 +/− 0.09 E2/E6 ratio) than EPE6 rafts (0.70 +/− 0.17 E2/E6 ratio, *P*<0.01) (Figure 4A). As well, E2 mRNA expression was significantly diminished in AAE6 (2.42×10^−4^ +/− 2.63×10^−4^) compared to EPE6 rafts (0.18 +/− 0.28, *P*<0.001) (Figure 4B), which could help explain the over-expression of E6/E7 in the AAE6 variant rafts. Although we cannot exclude integration occurring at sites other than the hinge-region of E2, and further work is required to definitively assess integration, these results are noteworthy as they provide a clue that there may be physical status differences between AAE6 and EPE6.

As an indicator of downstream effects of the E6 and E7 oncogenes' over-expression, we assayed the catalytic subunit of the human telomerase complex, hTERT. Tumourigenic HPVs, through the action of E6, are known to activate telomerase promotion, which subsequently aids in cellular immortalization [70]. Expression of hTERT, as evidenced by qRT-PCR, was significantly increased in AAE6 rafts (46.45 +/− 21.21 fold) compared to EPE6 (7.40 +/− 6.71 fold increase from NIKS, *P*<0.01) (Figure 4C). Both AAE6 and EPE6 rafts had significantly higher hTERT expression compared to NIKS (1.00 NIKS ratio, *P*<0.001 and *P*<0.01 respectively).

Our results suggest that, after 14 days in raft culture, the level of hinge-region E2-integrated viral copies (as assed preliminarily by E2/E6 ratios) increased more for AAE6 than EPE6, and the robustness of this finding was confirmed in three independent experiments with three biological replicates. Notably, significant E6, E7, E1∧E4, E2/E6 ratio, and E2 expression differences between variants were not detected in the monolayer cultures prior to rafting (*P*>0.05) [Figure S2], but only seen after 14 days of growth in the context of organotypic raft cultures. Thus, our three-dimensional model allows a relevant comparison to an *in vivo* scenario due to the potential for an active viral life cycle to take place. Taken together, and with previous results [59], we postulate that AAE6 may have an increased integration potential over EPE6, leading to its unique proliferation and differentiation patterns and potentially conferring persistence.

The mechanism of viral genome integration into the host genome, although still poorly understood, has been attributed to double-stranded DNA breaks and genomic instability enhanced by E6/E7-induced cellular proliferation [71]–[73]. This is exactly the scenario we observed, driven by the AAE6 genotype, with its enhanced proliferative ability, likely a cumulative result of an active Warburg effect [26], [27]. Initial changes in host keratinocytes are likely enacted by functional differences between AAE6 and EPE6, including increased proliferation capabilities, which has been demonstrated in E6-transduced PHFKs, with similar E6 expression in AAE6 and EPE6 cultures [27], [74]. It may be that AAE6 confers an increased rate of population doublings of host keratinocytes, resulting in increased genomic instability without cell cycle arrest, and ultimately a propensity toward integration. Although that is one possible explanation, it does not preclude the possibility that AAE6 protein has additional or alternative functional abilities that either increase genomic instability or decrease DNA damage response and DNA repair mechanisms. Additionally, since viral episome maintenance in primary keratinocytes is tightly controlled by E6 and E7 oncoproteins [75], our genomes that only contain AAE6 genotype changes (but not those in E7, for example) may be in part responsible for our observations. However, it is unclear how much of an impact this would have given our already immortalized keratinocytes, as E7 may not be necessary for episome maintenance after the fact [75].

High-risk HPVs, including type 16, are known to evade viral recognition by the host's innate immune system by modulating type 1 interferon (IFN) pathways [2], [76]. HPV16 E6 inhibits the transcriptional activity of interferon regulatory factor-3 (IRF-3), which can decrease type 1 IFN activation in infected keratinocytes [77]. Meanwhile, HPV16 E7 is known to repress the transcription of toll-like receptor 9 (TLR9), a dsDNA sensing molecule that is important for initiating an innate immune response [78]. Additionally, we and others previously reported the perturbed expression of a recently described, type 1 IFN, IFN-kappa (IFN-κ) [79]–[82], demonstrating its down-regulation by HPV16 in keratinocytes. In contrast, this type 1 IFN, which is constitutively expressed in keratinocytes, and elicits an anti-viral response via the type 1 IFN-stimulated transcription pathway, exhibited cell-associated anti-viral activity against a hepatitis C virus replicon cell line [83]. To further elucidate AAE6's persistence within infected cells and its downstream effects, we measured IFN-κ in our rafts cultures. IFN-κ was expressed in control and HPV16+ raft cultures, but was significantly decreased in AAE6 (0.22 +/− 0.06 NIKS ratio, 4.62 fold decrease) compared to NIKS (1.00 NIKS ratio, *P*<0.001) as well as in AAE6 compared to EPE6 (0.88 +/− 0.29 NIKS ratio, 1.12 fold decrease, *P*<0.001) (Figure 4D). IFN-κ was not statistically different between EPE6 and NIKS (*P*>0.05). A functional mechanism for E6-related IFN-κ silencing has been proposed to be epigenetic control by methylation [80]. As well, our observation of decreased IFN-κ expression along with increased p53 degradation is consistent with the suggestion that a positive feedback loop exists between the two [80]. Although more work is needed in the future to assess purely functional differences between the variant E6 proteins, it is probable the enhanced IFN-κ silencing in AAE6 is primarily related to the over-expression of E6 compared to the EPE6 variant in our experiments.

## Conclusion and Perspective

Throughout the years, our group has studied the immortalization and transforming potential of the E6 variants without and in the presence of E7 using monolayer and organotypic tissue cultures. Each time, AAE6 has demonstrated increased biological successes over EPE6 with regard to higher tumourigenic potential [25]–[27]. The present study builds on these previous findings and is the first to characterize the unique abilities of these two HPV16 E6 genotypes to drive tumourigenesis in host keratinocytes in the context of a full-length HPV16 genome and a three-dimensional epithelial raft model. To summarize our current findings in the context of existing knowledge, we suggest that the biological “success” of the AAE6 genotype over EPE6 is initiated by a variant-specific increase in proliferation due to the Warburg effect [26], [27], leading to a genetically unstable host environment [71]–[73]. Although only speculative, and requiring future experiments, this environment may selectively permit episomal loss and AAE6 viral integration into the host genome, resulting in an interruption and ultimate loss of E2 control over E6 and E7 expression [48]–[58]. The over-expression of E6 and E7 in AAE6-infected epithelia disrupts its typical differentiation pattern, resulting in dysplasia. This pre-malignant status has significant proliferative and tumourigenic effects within the host cell: p53 degradation, hTERT over-expression, and IFN-κ repression. Ultimately, our results supply a key link between the genotypic differences of AAE6 and the higher risk as well as the earlier occurrence of the AAE6 genotype for cervical tumours compared to EPE6 [20]. In addition, they provide important insight into the role that minor genomic variations can play in altering the transforming ability of tumourigenic viruses. We speculate that the selective pressures acting on the evolution of the AAE6 variant may have been unique from those acting on EPE6. Although definitive evidence is required to elucidate the role, if any, that integration may have, earlier integration into the host genome than EPE6 would paradoxically put AA at an evolutionary disadvantage by preventing further viral transmission. Counterbalancing by host population effects would be required to explain AAE6's continued prevalence. Further studies using the full variant genomes (including all variant SNP's as evolved via the process of lineage fixation) as well as a full suite of techniques are required to ultimately confirm our notion of AAE6-specific integration.

## Materials and Methods

### Cell culture

Mammalian cell cultures were maintained in a 5% CO_2,_ 37°C humidified incubator, with nutrient growth media replaced every second day and routine monitoring of *Mycoplasma* contamination, as indicated by DAPI-based testing. Normal or near-diploid immortalized keratinocytes (NIKS), which are capable of a normal differentiation regimen and can maintain the HPV genome episomally, were grown on mitomycin-C-treated Swiss mouse J2/3T3 fibroblast feeder layers, as described previously [28]. Primary human foreskin fibroblasts (ATCC CRL-2097) used for the dermal equivalent of organotypic raft cultures were only used at early-passage (less than five) and maintained in DMEM supplemented with 10% fetal bovine serum (FBS; Sigma-Aldrich, Cat. No. F6178) and 1% antibiotic/antimycotic (Invitrogen, Cat. No. 15240-062).

### Transfection of viral DNA into an immortalized cell line

Full-length viral DNA sequences for each HPV16 E6 genotype (EPE6 and AAE6) were created by site-directed mutagenesis (performed by GenScript) of the parental genome HPV16 W12E isolate (GenBank accession number AF125673) [38], [59], so that only the three non-synonymous nucleotide changes differentiated EPE6 and AAE6. EPE6 was created by changing nucleotide 350 from G to T. AAE6 was created by changing T to G at nucleotide 145 and C to T at nucleotide 335. These were then sub-cloned into the multiple cloning site of the high-copy number pUC19 (Fermentas) followed by sequencing of the vector ends, E6, E7, part of E1, part of L1, and the LCR was done as quality control to ensure no unintended mutations were introduced. Full-length E6 variant DNA (as an intact circular genome) was then generated via a previously described process of BamHI restriction digestion, gel electrophoresis purification and extraction, and T4 ligation followed by transfection into the immortalized NIKS cell line [29]. Since the re-ligated full-length HPV16 variant genomes contain no selective marker, a co-transfection strategy was employed using the ampicillin and neomycin resistant green fluorescent protein (GFP)-encoding pReceiver-M03 (GeneCopoeia). Thus, full-length genomes were transfected into NIKS with an equal amount (1 µg) of pReceiver-M03. FuGENE 6 Transfection Reagent (Roche Applied Science, Cat. No. 11814443001) was used 3∶1 to DNA (6 µL FuGENE reagent to 1 µg each of HPV16 and plasmid DNA). Under these conditions, it was theoretically possible that four general cell populations would exist: NIKS with neither plasmid, NIKS with HPV16 but no GFP/neo plasmid, NIKS with GFP/neo but no HPV16, and NIKS with HPV16 and GFP/neo plasmid. Under selective conditions, we expected at least half of the remaining population to have HPV16 DNA. HPV DNA presence was confirmed in monolayer transfectants by PCR while genome status and viral copy number were measured by qPCR-based assays as described below. Since our findings were within the range of a previously established 1 to 10 viral copy number per NIKS cell, confirmed by Southern blot [32], we determined that we successfully introduced full-length HPV16 genomes of EP and AA HPV16 variants into host keratinocytes.

### Establishment of organotypic "3D raft" cultures

Organotypic cultures were generated as previously described [25], [29], with minor technical modifications, as discussed below, to ensure reproducibility in our lab. We performed three independent raft experiments, each with three independent biological replicates (individual rafts, sample size *n* = 9). We determined through multiple trials that rat tail collagen type I [29] (rather than bovine collagen [38]) at a concentration between 3.75 and 4.00 mg/mL, was ideal for establishing an intact dermis. Early-passage human foreskin fibroblasts [29], when compared to high-passage pre-senescent cells, yielded the best epidermal growth. When creating the basal keratinocyte layer atop the dermis, we used media containing epidermal growth factor (EGF) to promote the activation of DNA transcription and a fully packed basal layer [28]. Thereafter, differentiation was induced by lifting the raft onto a membrane, forming an air-liquid interface. For differentiation of NIKS, media were supplemented with calcium [29] in the absence of EGF and fed through the bottom of each membrane in a 6 well-plate every second day for two weeks. Rafts destined for histological analysis were supplemented with 10 µM bromo-2-deoxyuridine (BrdU) for 24 hours prior to harvesting.

### Phenotypic characterization of rafts

Phenotypic characterization included confirming the presence and localization of HPV16 L2 capsid protein for active viral life cycle, BrdU for proliferation, K5/K10 for differentiation, p16^INK4A^ as a surrogate marker for E7 expression, and p53 for apoptosis. Rafts were harvested on day 14 for histological analysis, as described previously [25], [32]. Formalin-fixed, paraffin-embedded (FFPE) tissue slides were prepared by standard techniques [25]. Antigen retrieval was performed in either 0.01 M citrate (pH 6.0 for L2, BrdU, and K5/K10) or 0.01 M Tris Base with 0.001 M EDTA (pH 9.0 for p16^INK4A^ and p53), with a pressure cooker (Pascal, Dako Cytomation) at 125°C and 20 psi. Primary antibodies against HPV16 L2 (mouse mAb 1∶100, Gift from Dr. Martin Müller, #4D11), BrdU (mouse mAb 1∶100, Invitrogen, Cat. No. ZBU30), p16^INK4A^ (mouse mAb 1∶200, Santa Cruz Biotechnology, Cat. No. sc-56330), p53 (rabbit mAb 1∶50, DAKO, Cat. No. M3629), K5 (rabbit pAb 1∶10,000, Abcam, Cat. No. ab24647-50), and K10 (mouse mAb 1∶100, DAKO, Cat. No. M7002) were diluted in background-reducing diluent (Dako, Cat. No. S3022) and applied overnight at 4°C in a humidified chamber. Diluent was used as a negative technical control. Unbound primary antibody was removed by three successive 10 min chilled-PBS washes. Donkey anti-mouse secondary antibody-conjugated to Alexa Fluor 594 (1∶800, Life Technologies, Cat. No. A-21203) or donkey anti-rabbit secondary antibody-conjugated to Alexa Fluor 488 (1∶400, Life Technologies, Cat. No. A-21206) was applied for 30 min at room temperature in a dark, humidified chamber. Unbound secondary antibody was removed by three, 10 min chilled-PBS washes followed by dH_2_O rinses. Slides were mounted using Vectashield with DAPI (1.5 µg/mL, Vector Laboratories, Cat. No. H-1200).

Fluorescence was visualized and digitally captured using an inverted fluorescent microscope (Zeiss Axiovert 200 with mbq 52 ac power supply), digital camera attachment (QImaging QICAM Q21310), and Northern Eclipse software. For 350 nm emission (blue), 50 or 100 ms exposure time was used. For 488 nm (green) and 594 nm (red), 500 ms to 5.0 s exposure times were used, depending on target protein (kept consistent for all images of the same target). Images were captured with 1×1 binning, 1280×1024 resolution, 2.26% gain, 50.01% offset, and saved as “.bmp” or “.tiff”. Multiple fields of view were captured so as to have sufficient technical replicates to represent an entire raft. Post-capture modifications (such as overlays) were performed using ImageJ version 1.47a [84] and applied consistently to all images of the same target protein. Background fluorescence was subtracted by digital thresholding, followed by linear adjustments to brightness and contrast. The percentage of positively-stained cells (of total DAPI stained cells) was quantified for basal (bottom-most layer of cells) and suprabasal compartments using ImageJ's cell counter tool for each target protein. Technical replicates (fields of view) were averaged to generate a single datum per biological replicate (raft). For p16^INK4A^ quantification, only strong diffusely-stained cells were counted as positive [41].

### RNA expression profiling

Viral and host mRNA transcript expression was measured from organotypic raft cultures grown for two weeks (three independent raft experiments with three independent biological replicates each, sample size *n* = 9) using qRT-PCR to relatively quantify gene expression. First, residual medium was washed away with PBS, then sterile forceps were used to detach the epithelium and dermis into separate sterile nuclease-free 2 mL cryovials. Harvested samples were immediately flash-frozen using liquid nitrogen and stored at -80°C. RNA was extracted using the Arcturus PicoPure RNA Isolation Kit (Life Technologies, Cat. No. KIT0204), with the optional DNase treatment, and eluted in 30 µL of provided elution buffer. RNA quantity and integrity was assessed, reverse transcription to cDNA performed, and qRT-PCR set up as previously described by us [79]. Briefly, reactions consisted of 150 ng of cDNA, 45 µL of TaqMan Gene Expression Master Mix (Life Technologies, Cat. No. 4369016), 4.5 µL of TaqMan Gene Expression Assay hydrolysis probes (Table 1, see File S1 for custom assay standard curves), and nuclease-free dH_2_O to make up the volume to 90 µL. Triplicate reaction volumes of 25 µL were loaded into a transparent 96-well plate and analyzed. A positive cervical tumour control (to assess inter-plate variability), and a negative no-template control of nuclease-free dH_2_O, were run on each plate with the reference gene hypoxanthine phosphoribosyltransferase 1 (HPRT1). HPRT1 was chosen as a suitable reference gene based on previous optimization experiments [79]. Since the calibrator sample has zero expression, relative viral gene expression was calculated by the modified Livak method 2^−ΔCt^. For non-viral (host) gene expression, where the use of a calibrator sample (NIKS) was possible, the relative expression ratio (fold change) was calculated by the Livak method: 2^−ΔΔCt^ [85]. Both methods assume amplification efficiencies of the reference and target gene to be similarly close to 100%: *E* = 2.

**Table 1:** Gene expression assays used for qRT-PCR (Life Technologies) based on HPV16 EP reference sequence (GenBank accession number K02718).

TaqMan Gene Expression Assay	Assay ID	Forward Primer Sequence (5′ - 3′)	Reverse Primer Sequence (5′ - 3′)	Probe Sequence (5′ - 3′)	Start and Stop Position	Amplicon Length (bp)
HPRT1	Hs99999909_m1	N/A	N/A	N/A	N/A	100
hTERT	Hs00162669_m1	N/A	N/A	N/A	N/A	85
IFN-κ	Hs00737883_m1	N/A	N/A	N/A	N/A	108
*HPV16 E6	AIAAY0O	TGATATAATATTAGAATGTGTGTACTGCAAGCAA	GCATAAATCCCGAAAAGCAAAGTCA	CACGTCGCAGTAACTG	175–256	82
*HPV16 E7	AIBJW6W	GCTCAGAGGAGGAGGATGAAATAGA	GAGTCACACTTGCAACAAAAGGTT	CCAGCTGGACAAGCAG	653–749	97
*HPV16 E1∧E4	AIT9552	GTGTGCCCCATCTGTTCTCA	GCTGCCTAATAATTTCAGGAGAGGAT	TCCTGCAGCAGCAACG	829–880 ∧ 3357–3394	90
*HPV16 L2	AIFASOB	GCTCCAGATCCTGACTTTTTGGATA	ACCTAATGCCAGTACGCCTAGA	TAGGCCAGCATTAACC	5066–5141	76
**HPV16 E2	Separate probe and primer pair	AACGAAGTATCCTCTCCTGAAATTATTAG	CCAAGGCGACGGCTTTG	CACCCCGCCGCGACCCATA	3361–3442	82

N/A denotes sequence not available from Life Technologies.

*denotes custom-designed assay.

**denotes custom-designed assay, VIC instead of FAM probe.

∧denotes splice junction.

### HPV16 E2/E6 copy number assay

Raft DNA was extracted from deparaffinised FFPE tissue sections using a DNeasy Blood & Tissue Kit (Qiagen, Cat. No. 69504). Although this method of DNA extraction typically results in fragmented DNA (<650 bp), it was not expected to be problematic given our small PCR amplicons (∼100 bp) [86]. The E2/E6 copy number ratio was measured using a sensitive qPCR-based technique with a multiplex reaction for HPV16 E2 and E6 DNA copies, as previously described [69], [87]. E2-specific primers were used for the E2 hinge-region, which is commonly interrupted when integrated [65], [66]. Log-linear standard curves (log copy number per diploid cell versus cycle threshold, C_T_) were constructed from a 10-fold dilution series of known CaSki and SiHa E2 and E6 DNA copy numbers [88], [89]. As critically evaluated previously [89], CaSki has 869 E6 copies and 2,272 E2 copies per diploid cell while SiHa has 3.4 E6 copies and 0.7 E2 copies per diploid genome. Given that a typical human cell has a diploid genome mass of ∼6.6 pg (∼6×10^9^ base pairs in a diploid genome multiplied by an average of 1.096×10^−9^ pg per base pair), copy number per unit mass can be calculated and used to determine unknown copy number in samples when template mass is known (20 ng per reaction in our case). SiHa DNA was used as an E2 negative control. Intact E2 copies represent episomes (and potential non-E2 integration sites), while intact E6 alone represents episomal and E2-integrated forms. The E2/E6 copy number ratio, previously compared alongside traditional Southern blot [68], [69], [89], is a proxy for the average physical status of HPV16 genomes present in a sample: E2-integrated (E2/E6 ∼0), mixed (E2/E6 greater than 0 and less than 1), and episomal (E2/E6 greater than or equal to 1). However, this method has disadvantages in that it can only detect a subset of possible integrations (that is, ones that are in the E2-hinge region). Total HPV16 genome copy number was determined using the same method, but for the HPV16 L2 gene.

### Statistical analysis

All statistical analyses were performed using the statistical programming language R, version 3.0.1 [90]. Significance level (α) was set, *a priori*, at 0.05. Data were determined to be non-parametric based on parametric assumption violations of normality and homogeneity of variance. Normality was assessed subjectively using histograms and Q-Q plots, and objectively using Shapiro-Wilk's tests. Homogeneity of variance was assessed subjectively using boxplots, and objectively by Bartlett's or Levene's test. Non-parametric data, including comparisons between NIKS, EPE6, and AAE6, were subjected to non-parametric Kruskal-Wallis tests followed by pair-wise Wilcoxon rank-sum post-hocs with Bonferroni corrections for family-wise error. Wilcoxon rank-sum tests were used when comparing less than three group means for non-parametric data. Unless otherwise indicated, data are presented as means +/- standard deviation (SD).

## Supporting Information

Figure S1 **K5/K10 ratio as a measure of increasing basal phenotype.** Quantification of the ratio of K5 and K10 positive cells as a percentage of the total number of DAPI-stained nuclei in the raft epithelia of European Prototype (EP) HPV16 E6 variant (EPE6) and Asian-American (AA) HPV16 E6 variant (AAE6) cultures. Data are presented as means, while error bars represent SD. Statistical analyses performed were: a Kruskal-Wallis test followed by pair-wise Wilcoxon rank-sum post-hoc with Bonferroni correction (*n* = 4).
(TIF)

Figure S2 **Characterization of viral expression and status in monolayer cells prior to rafting.** Viral expression and physical status in European Prototype (EP) HPV16 E6 variant (EPE6) and Asian-American (AA) HPV16 E6 variant (AAE6) monolayer cultures. (**A, B, C, E**) Relative expression of E6, E7, E1∧E4, and E2 transcripts by qRT-PCR was calculated by the modified Livak method (2^−ΔCt^), since NIKS calibrator sample had zero viral gene expression. HPRT1 was used a reference gene. (**D**) E2/E6 ratio as an indication of genome status (episomal, mixed, or E2-integrated). Data are presented as means, while error bars represent SD. Statistical analyses of differences between EP and AA were performed by Wilcoxon rank-sum tests in all cases (*n* = 3 for all monolayer data).
(TIF)

File S1 **qRT-PCR standard curves for custom assays.** Standard curves (log_10_ copy number per diploid cell versus cycle threshold, C_T_) were constructed from a 10-fold dilution series of CaSki gDNA samples with known copy numbers. For the spliced transcript HPV16 E1∧E4 a cDNA sample from an EPE6 raft was used with a 5-fold dilution series of ng per reaction. Efficiency was calculated by using the following equation: (10^(-1/slope)^-1) ×100%.
(PDF)
