# Supplementary figures and images for: Tumourigenesis Driven by the Human Papillomavirus Type 16 Asian-American E6 Variant in a Three-Dimensional Keratinocyte Model
Source: PLoS One. 2014 Jul 1;9(7):e101540. doi: 10.1371/journal.pone.0101540 (PMC4077794; doi:10.1371/journal.pone.0101540)

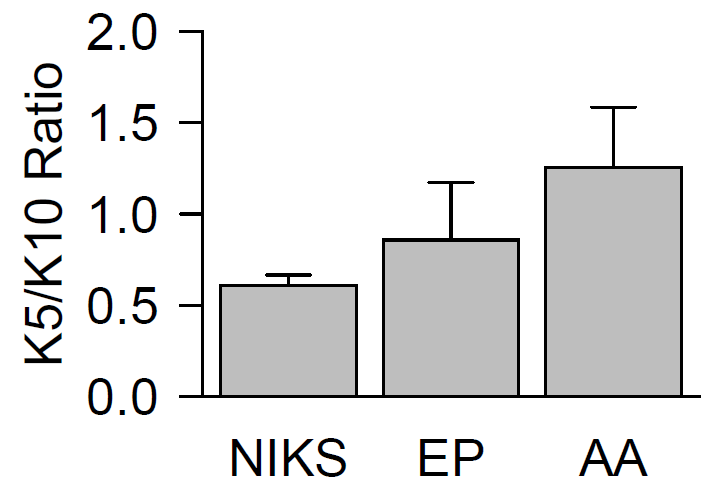

Supplement: Figure S1 — K5/K10 ratio as a measure of increasing basal phenotype. Quantification of the ratio of K5 and K10 positive cells as a percentage of the total number of DAPI-stained nuclei in the raft epithelia of European Prototype (EP) HPV16 E6 variant (EPE6) and Asian-American (AA) HPV16 E6 variant (AAE6) cultures. Data are presented as means, while error bars represent SD. Statistical analyses performed were: a Kruskal-Wallis test followed by pair-wise Wilcoxon rank-sum post-hoc with Bonferroni correction (n = 4). (TIF) [file pone.0101540.s001.tif]

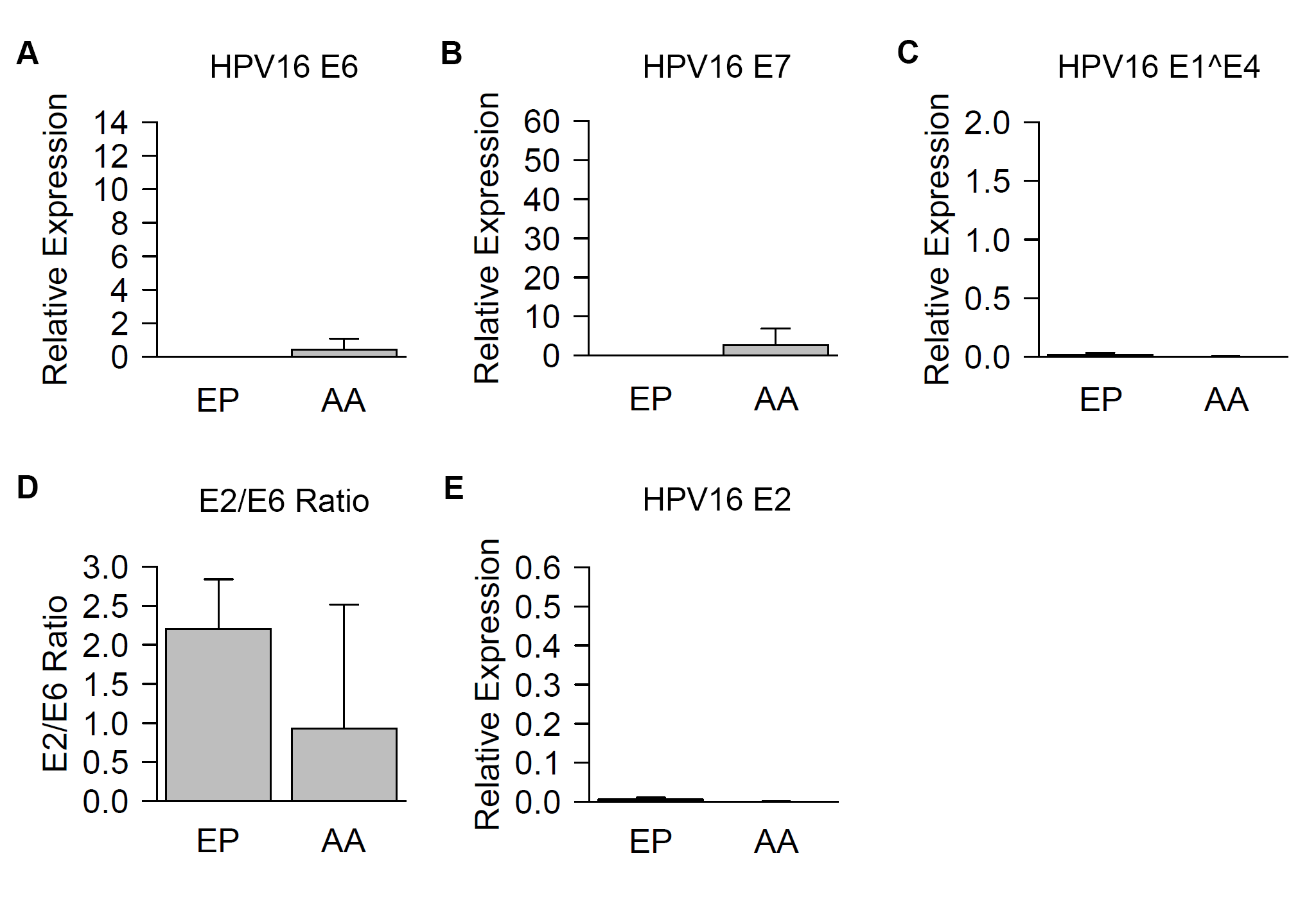

Supplement: Figure S2 — Characterization of viral expression and status in monolayer cells prior to rafting. Viral expression and physical status in European Prototype (EP) HPV16 E6 variant (EPE6) and Asian-American (AA) HPV16 E6 variant (AAE6) monolayer cultures. (A, B, C, E) Relative expression of E6, E7, E1∧E4, and E2 transcripts by qRT-PCR was calculated by the modified Livak method (2−ΔCt), since NIKS calibrator sample had zero viral gene expression. HPRT1 was used a reference gene. (D) E2/E6 ratio as an indication of genome status (episomal, mixed, or E2-integrated). Data are presented as means, while error bars represent SD. Statistical analyses of differences between EP and AA were performed by Wilcoxon rank-sum tests in all cases (n = 3 for all monolayer data). (TIF) [file pone.0101540.s002.tif]

### HPV16 E6 Std Curve

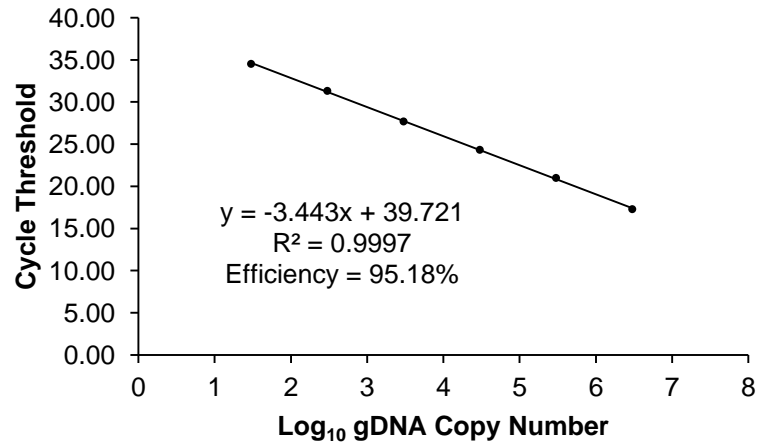

### HPV16 E7 Std Curve

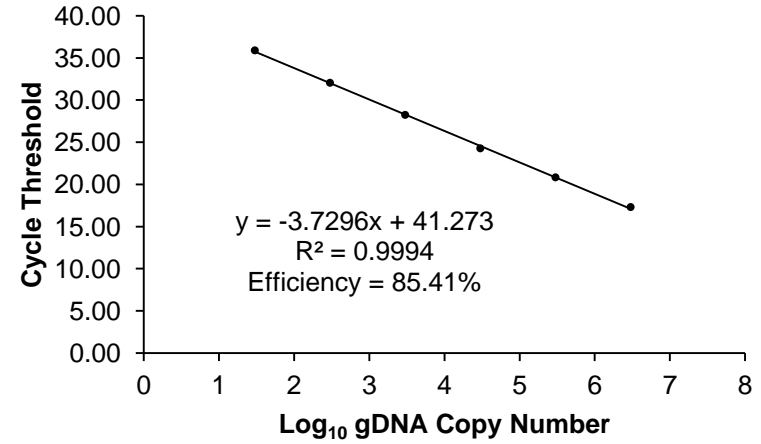

### HPV16 E2 Std Curve

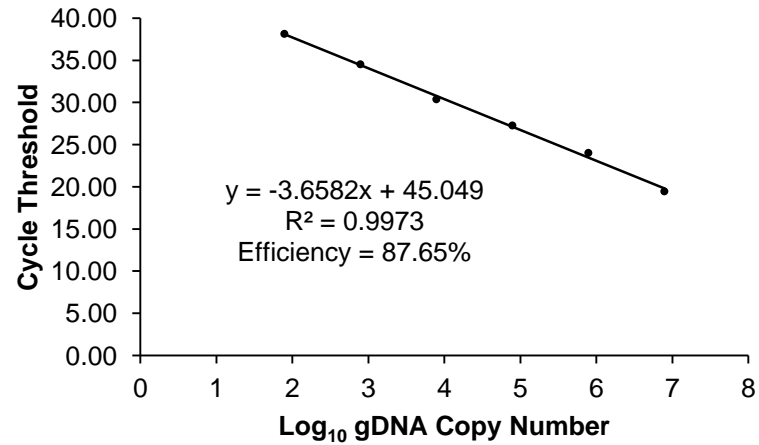

### HPV16 L2 Std Curve

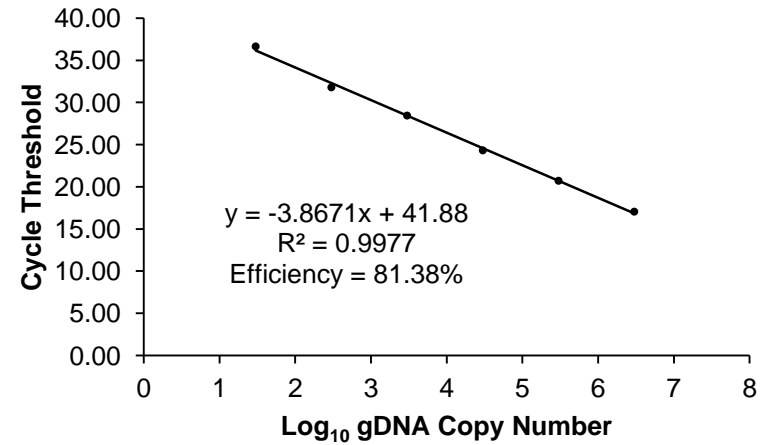

## HPV16 E1^E4 Std Curve

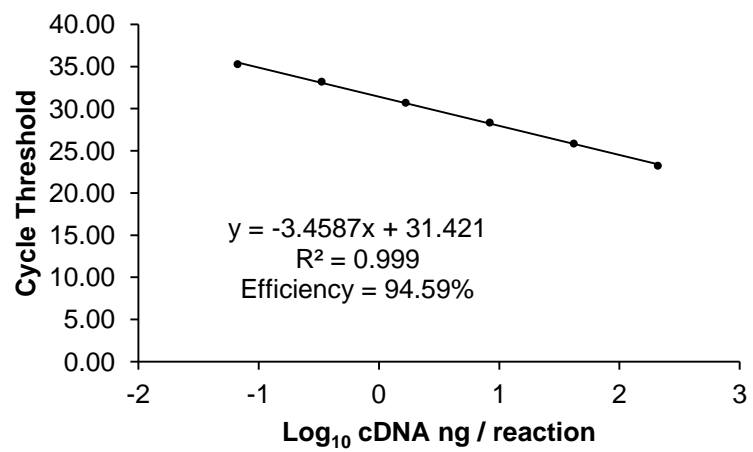

Supplement: File S1 — qRT-PCR standard curves for custom assays. Standard curves (log10 copy number per diploid cell versus cycle threshold, CT) were constructed from a 10-fold dilution series of CaSki gDNA samples with known copy numbers. For the spliced transcript HPV16 E1∧E4 a cDNA sample from an EPE6 raft was used with a 5-fold dilution series of ng per reaction. Efficiency was calculated by using the following equation: (10(-1/slope)-1) ×100%. (PDF) [file pone.0101540.s003.pdf]
